# Editorial: Hot topic: luminescence in rare earth coordination compounds

**DOI:** 10.3389/fchem.2023.1258607

**Published:** 2023-08-08

**Authors:** Paula Gawryszewska, Lawrence W. Miller, Artur J. M. Valente

**Affiliations:** ^1^ Faculty of Chemistry, University of Wroclaw, Wroclaw, Poland; ^2^ Department of Chemistry, University of Illinois Chicago, Chicago, IL, United States; ^3^ Department of Chemistry, CQC-IMS, University of Coimbra, Coimbra, Portugal

**Keywords:** rare earth elements, coordination compound, luminescence, biomedical, sensing

Rare-earth coordination compounds exhibit unique spectroscopic properties that include long emission decay times, high magnetic moments, narrow-bandwidth emissions, and large pseudo-Stokes shifts (Bünzli, 2015). These qualities underpin a wide array of photonic and biomedical technologies including light-emitting diodes [LEDs; (Kido and Okamoto, 2002)], anti-counterfeiting inks (Eliseeva and Bünzli, 2010), and chemical probes for diagnostics and bioimaging (Heffern et al., 2014). The ability of these metal ions to take part in luminescence resonance energy transfer (Qiu et al., 2022) and to exhibit photon upconversion (Wen et al., 2018) further expands their potential applications. Given their unique and fascinating properties, there is intense and ongoing interest in the fundamental and applied science of rare-earth coordination compounds and related molecular architectures, including metal-organic frameworks (Cui et al., 2014), polymer films, gels, hybrid materials (Binnemans, 2009), and nanomaterials (Wang et al., 2011).

The electronic configuration of rare-earth elements dictates their chemical and photophysical properties (Bünzli, 2015). Comprised of the lanthanide series, along with yttrium and scandium, the rare-earth elements are grouped together due to their similar geochemical properties. However, the only luminescent species are lanthanides with partially filled *4f* orbitals (elements 58–70) from cerium to ytterbium. Lanthanides are predominantly trivalent (Ln^3+^) and form cations in solution. The larger radial expansion of the 5s^2^5p^6^ subshells causes the *4f* orbitals to be shielded from the influence of their surroundings. Consequently, lanthanide coordination is omnidirectional and metal-ligand interactions are electrostatic.

A rigorous understanding of the physicochemical properties of lanthanides informs the design of functional coordination compounds. The orbital shielding results in very weak perturbations of *4f* electronic transitions by the ligand field that gives rise to lanthanides’ characteristically narrow, multi-line absorption and emission spectra. The *4f*–*4f* electronic transitions are electric dipole (ED) forbidden to first order because the initial and final states belong to the same parity. The types of intraconfigurational *4f–4f* electronic transitions that are allowed to first order are magnetic dipole (MD) or electric quadrupole (EQ) transitions (Tanner and Duan, 2010). The extinction coefficients (ε) of Ln^3+^ are typically small. This means that even if the compounds of these ions exhibit large values of intrinsic quantum yield ( $Q_{\mathrm{Ln}}^{\mathrm{Ln}}$ ), the brightness values (B) remain small (B = ε x $Q_{\mathrm{Ln}}^{\mathrm{Ln}}$ ). As a result, various ways to sensitize lanthanides emission are approached. One of them is the so-called antenna effect. It relies on the fact that the lanthanide ion is coordinated to organic ligands that have absorption transitions (e.g. π* ← π) with a high value of the extinction coefficient. After the absorption of electromagnetic radiation from the range of the ligand absorption band occurs, intramolecular non-radiative energy transfer from the ligand to the lanthanide ion takes place. Ln^3+^ are characterized by long decay times of the emission, in the order of micro- and milliseconds.

This Research Topic highlights the science of luminescent coordination compound design with four different contributions. The first article included in this Research Topic highlights the relevance of the structure of lanthanide complexes on luminescent properties. Luminescent processes based on the absorption of visible light and emission in the visible/NIR region are relevant for the development of biosensors for biomedical applications. Ohmagari et al. describe the syntheses of a highly planar tetradentate ligand and its corresponding complexes by using a set of different lanthanide ions. The resulting compounds show thermosensitive, visible light-excited, dual-color luminescence that reflects simultaneous ligand phosphorescence and metal *f-f* emission. The emission characteristics are a consequence of *π*-electronic system coplanarity through H-bonding, and the color profile of these complexes’ luminescence varies depending on the coordinated lanthanide ion. These features are discussed in terms of structural and photophysical properties.

The dinuclear lanthanide compounds containing hexafluoroacetylacetonate and pyrene-based phosphine oxide, an organic-based luminophore, are the core of the research paper by Nakai et al. With this complex, lanthanide coordination sets the geometry of two pyrene moieties into a stacked configuration that exhibits excimer emission. The identity of the coordinated lanthanide, Eu(III), or Gd(III) affects pyrene stacking geometry and emission spectra. Moreover, the compounds with coordinated Eu(III) provide a thermosensitive ratiometric luminescence based on 4f*→*4f* and excimer emission. The use of lanthanide coordination to control aggregation structure opens up new avenues in the design of luminescent materials.

Dinga et al. describe the development and characterization of spherical polymer particles (polymer beads) that contain Eu(III) complexes. Polymer particles with Eu(III) or other luminescent lanthanides have found widespread use as highly detectable labels for a variety of bioanalytical assays and point-of-care diagnostics. As discussed in the study, polymer beads incorporate up to hundreds of individual coordinated Eu(III) compounds, and their luminescence and biocompatibility require careful selection of coordinating ligands and appropriate surface functionalization. The authors analyze the luminescence properties of pure Eu(III) complexes with trifluoro-substituted, aromatic β-diketones, and TOPO (trioctylphosphinoxide) co-ligands as well as the complexes incorporated into polymer beads. Emission quantum yields of the obtained polymer beads for all coordination compounds are higher than 80%. The surface of the polymer bead with the best properties was functionalized and model proteins were coupled (Avidine, Neutravidine). The core-shell beads were tested for ELISA-like analyses and for lateral flow immunoassays, and high-end commercial beads were compared as control. It has been shown that obtained beads, taking advantage of large amplification factors and ultimate brightnesses, enable determinations in the attomolar regime and that they are superior to commercial materials.

The last contribution to this topical issue is a review of the thermal and photophysical properties of Eu^3+^ and Tb^3+^ coordination compounds with phenyl-containing carbacylamidophosphates. Materials containing Eu^3+^ and Tb^3+^ are important for red and green emissions, and their coordination compounds with β-diketones are the most studied. However, β-diketones are not good Tb^3+^ emission sensitizers. Hence, there is a search for sensitizers among other groups of ligands, and one of them is carboxamidophosphates. Kariaka et al. provide an illuminating discussion of photophysics and energy transfer processes in lanthanide light-converting molecular devices. A variety of carbacylamidophosphate coordination compounds were evaluated for their photophysical properties and the results were compared with those of β-diketonates.
